# Rac1 and Rac3 isoform activation is involved in the invasive and metastatic phenotype of human breast cancer cells

**DOI:** 10.1186/bcr1329

**Published:** 2005-09-30

**Authors:** Paige J Baugher, Lakshmi Krishnamoorthy, Janet E Price, Surangani F Dharmawardhane

**Affiliations:** 1 Molecular Cell and Developmental Biology Section and The Institute for Cellular and Molecular Biology, The University of Texas at Austin, University Station, Austin, TX 78712, USA; 2 Cancer Biology Department, The University of Texas MD Anderson Cancer Center, Holcombe Blvd, Houston, TX 77030, USA; 3 Universidad Central del Caribe, School of Medicine, P.O. Box 60327, Bayamon, PR 00960-6032, Puerto Rico

## Abstract

**Introduction:**

The metastatic progression of cancer is a direct result of the disregulation of numerous cellular signaling pathways, including those associated with adhesion, migration, and invasion. Members of the Rac family of small GTPases are known to act as regulators of actin cytoskeletal structures and strongly influence the cellular processes of integrin-mediated adhesion and migration. Even though hyperactivated Rac proteins have been shown to influence metastatic processes, these proteins have never been directly linked to metastatic progression.

**Methods:**

To investigate a role for Rac and Cdc42 in metastatic breast cancer cell invasion and migration, relative endogenous Rac or Cdc42 activity was determined in a panel of metastatic variants of the MDA-MB-435 metastatic human breast cancer cell line using a p21-binding domain-PAK pull down assay. To investigate the migratory and invasive potential of the Rac isoforms in human breast cancer, namely Rac1 and the subsequently cloned Rac3, we stably expressed either dominant active Rac1 or dominant active Rac3 into the least metastatic cell variant. Dominant negative Rac1 or dominant negative Rac3 were stably expressed in the most metastatic cell variant. Cell lines expressing mutant Rac1 or Rac3 were analyzed using *in vitro *adhesion, migration and invasion assays.

**Results:**

We show that increased activation of Rac proteins directly correlates with increasing metastatic potential in a panel of cell variants derived from a single metastatic breast cancer cell line (MDA-MB-435). The same correlation could not be found with activated Cdc42. Expression of a dominant active Rac1 or a dominant active Rac3 resulted in a more invasive and motile phenotype. Moreover, expression of either dominant negative Rac1 or dominant negative Rac3 into the most metastatic cell variant resulted in decreased invasive and motile properties.

**Conclusion:**

This study correlates endogenous Rac activity with high metastatic potential and implicates Rac in the regulation of cell migration and invasion in metastatic breast cancer cells. Taken together, these results suggest a role for both the Rac1 and Rac3 GTPases in human breast cancer progression.

## Introduction

Cancer metastasis is a multi-faceted process requiring the disregulation of numerous signaling pathways, including those associated with cell adhesion and motility. The initial steps of metastasis require the acquisition of a motile phenotype in order to traverse tissue boundaries, while the later stages require dynamic adhesive interactions with the extracellular matrix to facilitate the extravasation of malignant cells [1]. Activation of the Rho family GTPases Rac and Cdc42 is a critical event in the integrin and growth factor-mediated regulation of cellular migration and adhesion, implicating the hyperactivation of these proteins in the progression of metastatic disease [2].

Activation of Rac and Cdc42 is critical for initiating cell motility and adhesion via the dynamic turnover of cell-substratum contacts (focal adhesions) and the nucleation of actin monomers necessary for the assembly of actin filaments required for cell movement [2]. Activation of the appropriate levels of these proteins, together with temporal and spatial coordination, must be precisely regulated to achieve normal cellular function [3]. Aberrant Rac and Cdc42 activities have been recently associated with invasive and malignant behavior in a variety of cell types, including hepatocarcinoma, breast carcinoma, and melanoma [4-6]. Breast tissue sample analysis has demonstrated, however, that the contribution of the Rac and Cdc42 proteins to tumor cell invasion in breast cancer is not due to genetic mutation, but is due instead to changes in the activity levels of these proteins caused by hyperactivation of upstream activators [3,7]. Yet, a direct correlation between Rac and Cdc42 protein activity states and metastatic progression in human breast cancer remains to be demonstrated.

The Rac subfamily includes Rac1, the myeloid-lineage specific Rac2, and the subsequently cloned Rac3 protein [8]. Exhibiting an 89% and 92% identity to Rac1 and Rac2, respectively, Rac3 differs from other Rac proteins only in the carboxyl terminus, a region essential for subcellular localization and regulatory protein binding [8,9]. In fact, differential localization of Rac1 and Rac3 has been demonstrated in the developing mammalian brain [10]. Moreover, dominant activation of Rac3 in the mammary epithelium has been shown to lead to the formation of mammary lesions [11], although a direct role for Rac3 in breast cancer invasion and metastasis has yet to be substantiated.

To further understand the molecular mechanisms of the small GTPases Rac and Cdc42 in human breast cancer, we used a panel of cell variants, isolated from the MDA-MB-435 human metastatic breast cancer cell line, that varied in their ability to form secondary pulmonary and cerebral lesions in the nude mouse model of experimental metastasis [12]. Within this panel, we found a direct correlation between the invasive phenotype, enhanced migratory ability, and increased metastatic potential. Moreover, we found that increased Rac, but not Cdc42, activation correlated with increased metastatic potential.

Previously, Rac1 was shown to play a critical role in rat mammary tumor cell growth and metastasis *in vivo *[5]. To establish a role for both Rac1 and Rac3 in human breast cancer, we carried out a comparative study of the invasive capabilities between the two isoforms. Dominant active Rac1 or Rac3 mutants were expressed in the least metastatic cell variant of our panel, and dominant negative Rac1 or Rac3 mutants were expressed in the most metastatic cell variant. Dominant active Rac expression of either isoform resulted in an aggressive phenotype, as well as significant increases in adhesion to extracellular matrix, migration, and invasion through basal lamina. Conversely, dominant negative expression of either isoform resulted in significant decreases in adhesion to extracellular matrix, migration, and invasion. Taken together, these data suggest a direct role for both Rac1 and Rac3 protein activation in the metastatic progression of human breast cancer.

## Materials and methods

### Cell culture

The human breast cancer cell line variants MDA-MB-435α6HG6, MDA-MB-435, MDA-MB-435α6LF9, and MDA-MB-435Br1 were cultured in Dulbecco's modified Eagle's medium (Gibco™, Carlsbad, CA, USA) with 10% v/v FBS (Tissue Culture Biologicals, Tulare, CA, USA) and cultured in a humidified 5% CO_2 _atmosphere at 37°C.

### DNA constructs and transfections

Rac1 mutant cDNA (Myc-Rac1(G12V) and Myc-Rac1(T17N)) were generous gifts from Dr Gary Bokoch of the Scripps Institute (La Jolla, CA, USA). Rac3 mutant cDNA (Myc-Rac3(G12V) and Myc-Rac3(T17N)) were generous gifts from Dr Ulla Knaus of the Scripps Institute (La Jolla, CA, USA). Mutant Rac cDNAs were digested out of the pRK5myc vector and inserted into the multiple cloning site of the pIRESneo2 vector (Clontech, Mt. View, CA, USA). pIRESneo2 vector alone, or vectors encoding myc-tagged Rac1(G12V), Rac1(T17N), Rac3(G12V), or Rac3(T17N) were transfected into cell variants using Lipofectamine Plus Reagent (Gibco™). Maximal expression was achieved 24 to 48 h post transfection with a transfection efficiency of approximately 70% at 48 h, as monitored by staining for myc expression. All experiments were conducted at 36 h following transfection and confirmed using stable cell lines.

### Immunofluorescence microscopy

Cells cultured on glass coverslips were fixed in 3.7% formaldehyde (Sigma Chemical Corp., St. Louis, MO, USA), permeablized with 0.5% Triton X-100 (Sigma Chemical Corp.), and blocked with 5% goat serum (Gibco™) and 5% BSA (Sigma Chemical Corp.). Cells were then stained with rhodamine phalloidin (Molecular Probes, Eugene, OR, USA) to visualize F-actin, and a mouse monoclonal anti-phosphorylated tyrosine antibody, clone 4G10 (Upstate Biotechnology, Waltham, MA, USA), followed by fluorescein isothyocianate (FITC)-conjugated goat anti-mouse IgG (ICN Biomedicals Inc., Irvine, CA, USA) as in [13]. Cells were imaged with either an Olympus (Hamburg, Germany) upright fluorescence microscope or a Zeiss (Thornwood, NY, USA) inverted confocal microscope with fluorescence and DIC (Differential Interference Contrast) capabilities. Images were overlayed with Spot Advanced digital camera software (Diagnostic Instruments Inc., Sterling Heights, MI, USA).

### Adhesion assays

Cell adhesion assays were performed according to Klemke *et al*. [14]. Briefly, glass coverslips (Fisher Scientific, Pittsburg, PA, USA) were coated with laminin (Gibco BRL, Carlsbad, CA, USA). Proteins were allowed to bind overnight at 4°C before the coverslips were blocked for 1 h with 1% w/v heat-denatured BSA (Sigma Chemical Corp.) in 1× PBS. Cells (10^5^) were added to the wells and allowed to adhere for 15 minutes. Non-adherent cells were removed, and the adherent cells were fixed in 3.7% formaldehyde (Sigma Chemical Corp.). The number of cells per microscopic field for 30 fields per coverslip was counted with an Olympus upright microscope with a 40× phase contrast objective. Non-specific cell adhesion as measured on BSA-coated coverslips has been subtracted. Effects of the ectopic expression of Rac mutants were assessed 36 to 48 h post transfection.

### Haptotaxis migration and invasion assays

Cell migration and invasion assays were performed as described in Klemke *et al*. [14]. Briefly, modified Boyden chambers (tissue culture treated, 6.5 mm diameter, 10 μm thickness, 8 μm pores, Transwell^®^, Costar Corp., Cambridge, MA, USA) containing polycarbonate membranes were coated with matrigel (Fisher Scientific) or laminin (Gibco BRL) on the underside of the membrane (migration), or the upperside of the membrane (invasion). For invasion assays, cells chemotracted to media supplemented with 10% v/v FBS (Tissue Culture Biologicals). Serum starved cells (10^6 ^cells) were added to the upper surface of each migration chamber and allowed to migrate to the underside of the membrane for 4 h (migration) or 24 h (invasion). The non-migratory cells on the upper membrane surface were removed, and the migratory cells attached to the bottom surface of the membrane were stained with propidium iodide (PI) (CalBioChem-Novabiochem Corp., San Diego, CA, USA). For PI staining, cells were fixed and permeablized in 70% ethanol and then incubated with 40 μg/ml PI in 1× PBS. The number of migratory cells per 30 microscopic fields per membrane was counted with an Olympus upright fluorescence microscope with a 40× objective for migration assays and a 10× objective for invasion assays. Non-specific migration as measured on chambers with no chemoattractant has been subtracted. Effects of the ectopic expression of Rac mutants were assessed 36 to 48 h post transfection.

### Rac and Cdc42 activity assays

For guanine nucleotide binding, cell lysates were incubated for 15 minutes at 30°C in the presence of 10 mM EDTA and 100 μM GTPγS (Roche, Rockford, IL, USA) or 1 mM GDP (Sigma) to facilitate nucleotide exchange as described in Knaus *et al*. [15]. The loading reaction was stopped by the addition of MgCl_2_.

Rac and Cdc42 activity assays were performed as described in [16] with minor modifications. Briefly, cells were lysed directly in their 10 cm plates (Fisher Scientific) with ice cold lysis buffer. Lysates were then incubated at 4°C with 10 μg of PAK-PBD Protein GST Beads (Cytoskeleton Inc., Denver, CO, USA). The bead pellet was washed once with buffer containing 1% Nonidet P-40 (Calbiochem, San Diego, CA, USA), twice without Nonidet P-40, and suspended in 20 μl Laemelli sample buffer. Proteins from the total cell lysate, as well as the bead pellet, were separated by 10% SDS-PAGE gel, transferred to a nitrocellulose membrane, and blotted for the appropriate GTPase using a monoclonal anti-Rac antibody (clone 32A8; Upstate Biotechnology) or a mouse monoclonal anti-Cdc42 antibody (clone 44; Transduction Laboratories, San Diego, CA, USA). Immunoblots were detected with the SuperSignal West Femto-Substrate chemiluminescence kit (Pierce Endogen, Rockford, IL, USA) and Kodak Biomax MR film (Fisher Scientific).

### Toxin B Inhibition

*Clostridium difficile *toxin B was purchased from Calbiochem and used as described in [17,18]. Cells were treated with 2 ng/ml toxin B for 24 h before being subjected to haptotaxis assay. These were the conditions required for suppression of all Rho GTPase activity while maintaining 95% cell viability.

### Statistical analysis

Data are expressed as means ± standard error of the mean (SEM). *P*-values were calculated from unpaired or paired t-tests using Microsoft Excel (Microsoft Corp., Redmond WA, USA) and considered significant at *p*-values less than 0.05.

## Results

### Cytoskeletal and migratory phenotype of MDA-MB-435 metastatic variants correlates with metastatic efficiency

To understand the role of the Rho GTPases in metastatic breast cancer, we selected variants of the MDA-MB-435 metastatic breast cancer cell line and cycled them through the nude mouse model of experimental metastasis. The variants studied were chosen based on their ability to metastasize from tumors in the mammary fatpad to distant organs, predominantly lungs and lymph nodes. The MDA-MB-435α6HG6 variant produced the highest number of distant metastases from primary mammary tumors, followed by the parental MDA-MB-435, then MDA-MB-435α6LF9, and finally MDA-MB-435Br1. The MDA-MB-435Br1 cell line readily forms experimental brain metastases following injection into the intra-carotid artery, yet it shows significantly lower ability to metastasize in the more stringent assay of spontaneous metastasis [12].

**Figure 1:**
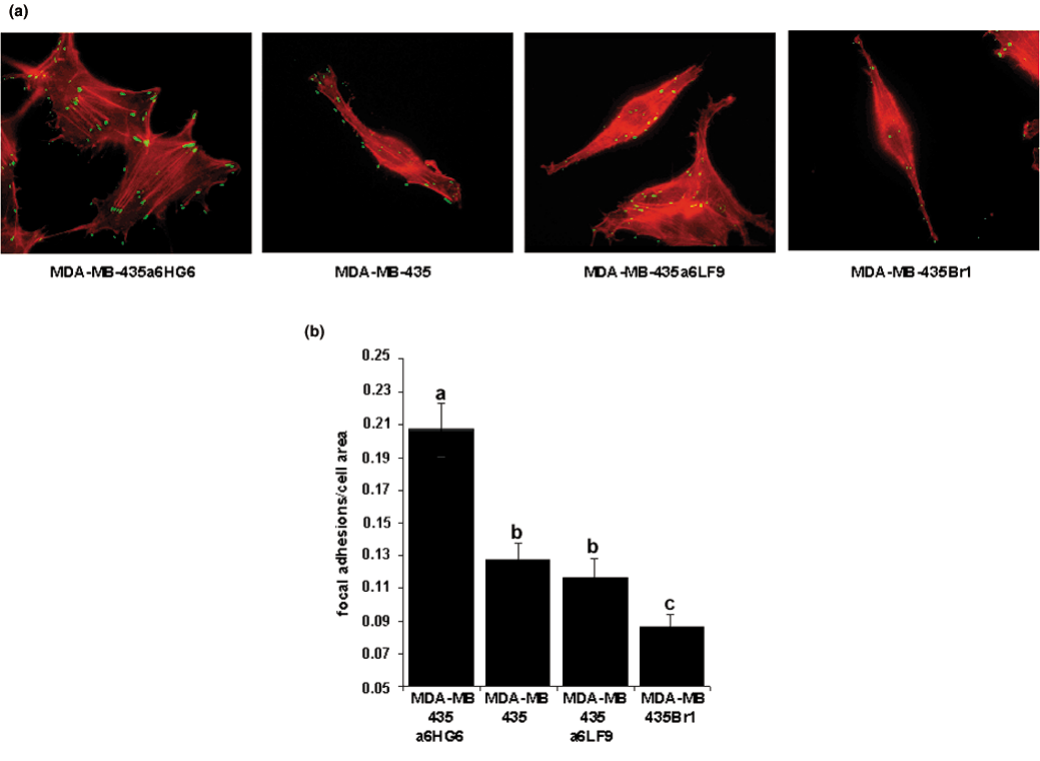
Characterization of cytoskeletal structures and focal adhesion distribution in MDA-MB-435 metastatic variants. **(a) **Each of the MDA-MB-435 metastatic variants was plated onto glass coverslips. Actin was visualized with rhodamine phalloidin and focal adhesions were visualized with an anti-p-tyro antibody followed by an FITC conjugated secondary antibody. **(b) **Cell area was measured on 50 individual cells per variant using Spot Digital Camera Software. Focal adhesion number was divided by cell area and plotted on the y-axis. Bars represent ±SEM, and are representative of three independent experiments. Treatments denoted by the same letter indicate no significant difference between those treatments. Treatments denoted by different letters indicate a significant difference between those treatments (*P *< 0.01).

An invasive cellular phenotype can be indicative of metastatic behavior [14]. Rac-induced membrane ruffles, or lamellipodia, have been shown not only to be important structures in cellular motility, but have also been shown to play a key role in invasion with respect to metastatic progression [2,19,20]. Rac-induced lamellipodia contain many cell-substratum contacts, or focal adhesions. Aberrant focal adhesion expression has been associated with malignant progression [21]. Therefore, we investigated the correlation between cytoskeletal phenotype and metastatic efficiency. Our data show a direct correlation between high lamellipodia expression and increased metastatic efficiency (Fig. 1a). The most metastatic variant, MDA-MB-435α6HG6, exhibits a strikingly different phenotype than the other variants, including an increased number of focal adhesions and a cross-linked actin network. In fact, we found that increased focal adhesion expression correlates with increased metastatic efficiency in all cell variants (Fig. 1b). However, individual MDA-MB-435α6HG6 (most metastatic) cells were 1.5 times larger than other variants (data not shown). Thus, the data were compiled as focal adhesions per cell area (Fig. 1c). This correlation between lamellipodia, focal adhesions, and metastatic potential strongly suggests an enhanced invasive and motile phenotype correlates with increased metastatic efficiency.

**Figure 2:**
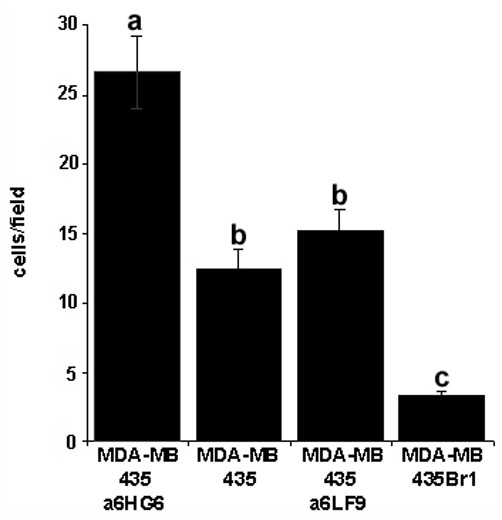
Haptotaxis assays of MDA-MB-435 metastatic variants. Each variant was adjusted to 500,000 cells and applied to Transwell chambers in a haptotaxis assay, where the underside of the membrane was coated with matrigel. Cells migrating to the underside of the chamber were stained with propidium iodide and counted under 400× Magnification. Bars represent ±SEM. Data are representative of three independent experiments. Treatments denoted by the same letter indicate no significant difference between those treatments. Treatments denoted by different letters indicate a significant difference between those treatments (*P *< 0.01, calculated from unpaired t-test).

Subsequent to the epithelial to mesenchymal transition, cells must first migrate away from the primary tumor through the basal lamina to begin the process of establishing sites of secondary tumor formation. Therefore, increased cell migration in malignant cells is thought to be closely linked to invasion and metastasis [1,2]. Upon investigation into the migratory behavior of the cell variants, we found a correlation between increased metastatic potential and increased migration (Fig. 2).

### Increased Rac activity can be correlated with MDA-MB-435 metastatic variants

**Figure 3:**
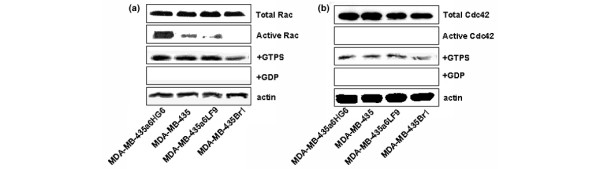
Rac and Cdc42 activity in MDA-MB-435 metastatic variants. Whole cell lysates of all variants were subjected to SDS-PAGE followed by western blot analysis for **(a) **total Rac using an anti-Rac antibody and **(b) **total Cdc42 using an anti-Cdc42 antibody. Rac and Cdc42 activities were assayed using the PAK-PBD activity assay. A non-hydrolyzable GTP analog, GTPγS, was used as the positive control; GDP alone was used for the negative control. Equal loading of lanes was maintained by performing a total protein assay and is confirmed by western blot analysis for total actin. Results are representative of three to five independent experiments.

Increases in activity levels of the Rho proteins Rac and Cdc42 have been shown to be accountable for the promotion of tumor cell invasiveness [3,7]. Therefore, we investigated the Rac and Cdc42 activity levels in all MDA-MB-435 metastatic variants. To determine the relative amounts of activated Rac and Cdc42 in the variant panel, we used Rac and Cdc42 activity assays [16]. Whereas total endogenous Rac protein expression remains equal among the cell variants, levels of Rac protein activity ranged from being highest in the most metastatic variant, medium in parental and medium metastatic variants, to least active in the least metastatic variant (Fig. 3a). Loading cell lysates with a non-hydrolyzable GTP analog, GTPγS, showed a relatively equal GTP-binding capacity of the Rac protein among the four variants. Therefore, all Rac expressed in the variants of the metastatic panel can potentially be activated to the same extent. Thus, endogenous activators of Rac appear to have increased activity in the more metastatic cell variants. Endogenous Cdc42 protein expression differed slightly among the variants; the more metastatic variants expressed slightly higher levels of endogenous Cdc42 than the less metastatic variants (Fig. 3b). However, no active Cdc42 protein could be detected. Again, GTPγS loading showed the ability of the Cdc42 proteins to bind GTP and become active.

### Inhibition of Rho GTPase activity suppresses invasive potential of MDA-MB-435 cells

**Figure 4:**
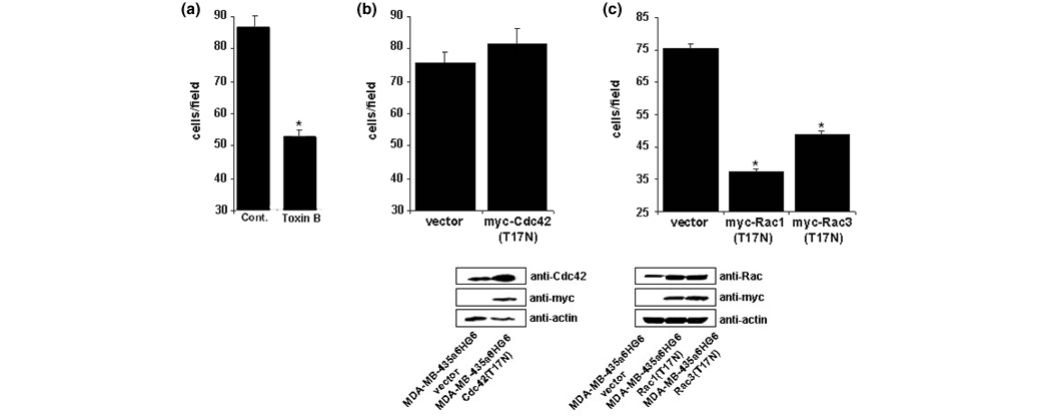
Inhibition of Rho GTPases, Rac, or Cdc42 in migration of the high metastatic MDA-MB-435α6HG6 cell variant. **(a) **MDA-MB-435α6HG6 cells were treated with vehicle or 2 ng/ml toxin B for 24 h and subjected to a basement membrane haptotaxis assay. Cells migrating to the underside of the membrane were stained with propidium iodide and counted under 400× Magnification. **(b) **MDA-MB-435α6HG6 cells transiently expressing vector alone or myc-Cdc42(T17N) were subjected to a basement membrane haptotaxis assay. Cells migrating to the underside of the membrane were stained with propidium iodide and counted under 400× Magnification. Equal loading was confirmed by a total actin blot, ectopic myc-Cdc42(T17N) expression confirmed by western blotting with anti-Cdc42 or anti-myc. **(c) **MDA-MB-435α6HG6 cells transiently expressing vector alone, myc-Rac1(T17N), or myc-Rac3(T17N) were subjected to a basement membrane haptotaxis assay. Bars represent ±SEM; equal loading was confirmed by total actin blot. Myc-Rac1(T17N) and myc-Rac3(T17N) expression were confirmed by western blotting with anti-Rac or anti-myc. Data are expressed as mean ±SEM of three independent experiments. A star denotes statistical significance from control (*P* < 0.05, calculated from paired t-tests).

Because the Rho family of small GTPases, namely Rho, Rac, and Cdc42, are essential to cell motility, we used *Clostridium difficile *toxin B to inhibit the Rho family in these cell variants. Toxin B was used as described in [17,18]. Inhibition of Rho GTPases by incubation in 2 ng/ml toxin B for 16 h demonstrated the expected attenuation of Rac-induced lamellipodia in response to epidermal growth factor. Subsequent to treatment with toxin B, the most metastatic variant, MDA-MB-435α6HG6, exhibited a 50% decrease in migration to basal lamina, and the others exhibited a definite decrease in migration, although not as striking (Fig. 4a). Therefore, increased migration in the most metastatic variant appears to be regulated in part by the Rho family of small GTPases.

Hyperactive Cdc42 has been implicated in tumor cell invasion due to its effects on the actin cytoskeleton [5,20]. To determine a role for Cdc42 in the migration of highly metastatic cells, we expressed vector alone and a dominant negative myc-tagged Cdc42(T17N) construct in the highly metastatic MDA-MB-435α6HG6 cell variant and subjected both to a migration assay. We found that expression of Cdc42(T17N) did not inhibit migration compared to the vector alone control (Fig. 4b). However, when we expressed vector alone, dominant negative Rac1(T17N), or dominant negative Rac3(T17N), we found a significant inhibition of migration compared to the vector control (Fig. 4c). Therefore, Rac activity appears to be essential for the migration of highly metastatic cells, but Cdc42 activity does not.

### Ectopic Rac(G12V) expression augments the invasive phenotype of low metastatic breast cancer cells

**Figure 5:**
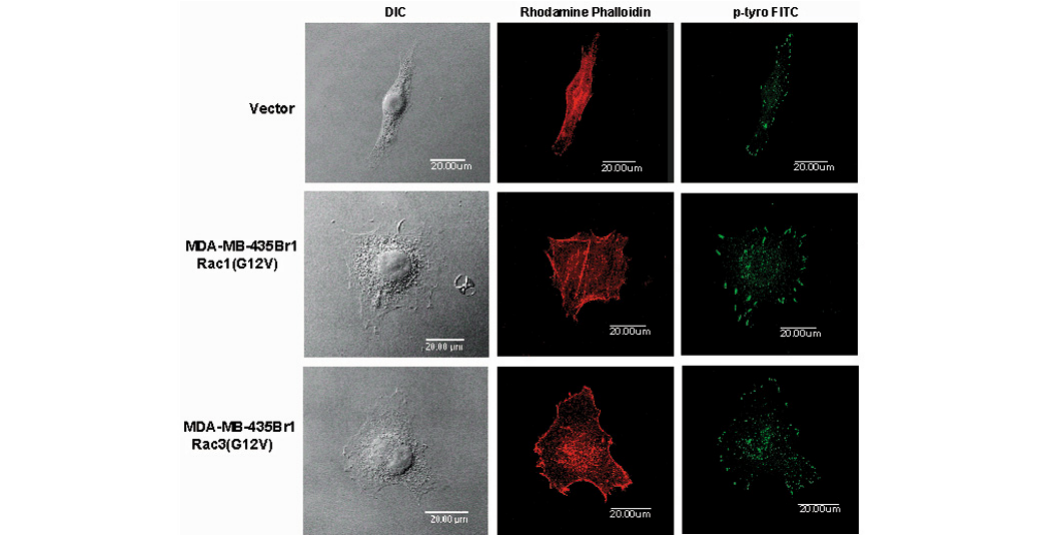
Ectopic dominant active Rac(G12V) expression in low metastatic variant MDA-MB-435Br1. Confocal DIC (Differential Interference Contrast) and fluorescent microscopy was performed on MDA-MB-435Br1 cell variant stably expressing vector alone, myc-Rac1(G12V), or myc-Rac3(G12V). Cells were plated on glass coverslips, fixed in 3.7% formaldehyde and permeablized with 0.2% Triton X-100. Actin was then visualized with rhodamine phalloidin and focal adhesions were visualized with an anti-p-tyro antibody followed by an FITC conjugate.

Invasive malignant cell morphology includes an increased number of focal adhesions, as well as an increase in actin structures such as cross-linked actin fibers and membrane ruffles [1,2]. The morphology of the low metastatic cell variant MDA-MB-435Br1 when expressing vector alone is indicative of a less invasive cell. Actin fibers are not cross-linked, lamellipodia are limited to the proximal and distal ends of the cell, and focal adhesions are few (Fig. 5). Conversely, MDA-MB-435Br1 cells expressing myc-tagged Rac1(G12V) or Rac3(G12V) exhibit cross-linked actin fibers, numerous focal adhesions, and lamellipodia expressed ubiquitously around the periphery of the cell (Fig. 5). Expression of either myc-tagged Rac1(T17N) or Rac3(T17N) in the highly metastatic MDA-MB-435α6HG6 variant resulted in a slight decrease in focal adhesion number and size of lamellipodia compared to the vector alone control (data not shown). Expression of dominant negative Cdc42(T17N) in the same cell variant resulted in no significant alteration of phenotype compared to the vector alone control (data not shown).

### Rac mutants significantly alter cellular processes essential to metastatic behavior

Because metastatic progression results from increased migration of malignant cells out of the basal lamina, subsequent adhesion to the extracellular matrix, and final invasion into distant tissues to establish secondary sites of metastasis, we measured the effect of Rac mutants on these processes *in vitro*. For each of these assays, cells expressed equal amounts of activated Rac1 or Rac3 mutant protein (data not shown).

**Figure 6:**
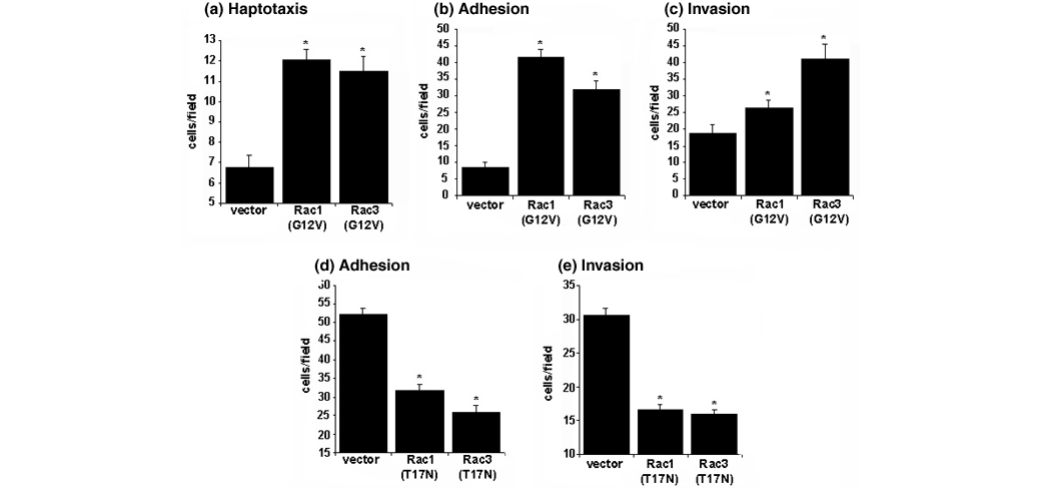
Effects of mutant Rac isoforms on metastatic properties *in vitro*. MDA-MB-435Br1 cells transiently transfected with vector alone, myc-Rac1(G12V), or myc-Rac3(G12V), were subjected to **(a) **adhesion, **(b) **haptotaxis, and **(c) **invasion assays. MDA-MB-435α6HG6 cells transiently expressing vector alone, myc-Rac1(T17N), or myc-Rac3(T17N), were subjected to **(d) **adhesion, and **(e) **invasion assays. Cells were counted under 200× Magnification for adhesions assays, and 400× Magnification for haptotaxis and invasion assays. Y-axis represents the number of cells/field for at least 20 microscopic fields per variant. Bars represent standard error of the mean, and are representative of at least three separate experiments. An asterix indicates a statistically significant difference compared to the control, vector alone, as determined by a paired Student's *t*-test (*P *< 0.05).

Recent data indicate that changes in cell adhesion play a critical role in tumor progression [21]; thus, we tested the ability of Rac mutants to alter adhesive properties of malignant cells *in vitro*. Expression of dominant active Rac1(G12V) or Rac3(G12V) causes a significant increase in adhesion to basal lamina when expressed in the low metastatic MDA-MB-435Br1 variant compared to the vector alone control, whereas dominant negative Rac1(T17N) or Rac3(T17N) cause a significant decrease in adhesion when expressed in the high metastatic MDA-MB-435α6HG6 variant (Fig. 6a,d).

A requirement of malignant cells to undergo metastasis is to acquire the ability to penetrate the surrounding extracellular matrix in order to migrate to distant tissues [22]; thus, we tested the effect of Rac mutants on both migration and invasion *in vitro*. Expression of either myc-tagged Rac1(G12V) or Rac3(G12V) caused a significant increase in migration and invasion when expressed in the low metastatic variant (Fig. 6b,c). Surprisingly, Rac3(G12V) expressing cells invaded through basal lamina 1.5 times more than cells expressing Rac1(G12V); however, this difference was a trend and not statistically significant (Fig. 6c). Invasion of highly metastatic cells expressing dominant negative Rac1(T17N) or Rac3(T17N) was significantly diminished compared to the vector alone control (Fig. 6e). Furthermore, migration was also significantly reduced in highly metastatic cells expressing dominant negative mutants of Rac isoforms compared to vector alone control (Fig. 3b). Taken together, these data establish the efficacy of both Rac1 and Rac3 in human breast cancer progression.

## Discussion

The present study demonstrates a strong correlation between the invasive phenotype, increased Rac activity, and the increased metastatic efficiency of the variants of the MDA-MB-435 metastatic human breast cancer cell line. These cell variants reflect the relative ability to form lung metastases from established mammary tumors in the nude mouse model of experimental metastasis.

The invasive phenotype, characterized by extensive cross-linked actin networks and increased lamellipodial expression, has long been associated with increased motility and invasion [23]. More recently, this specific phenotype has been linked to cells with an inherent ability to metastasize [1,2]. Our study adds to this field by correlating an increasingly aggressive phenotype with an increase of metastatic efficiency in a panel of metastatic cell variants. Oncogenically mutated forms of Rac and Rho proteins have not been found in human cancer cells; instead, it is thought that amplification of Rho family proteins or activation of their upstream regulators, such as exchange factors, contribute to the ability of these GTPases to influence the transformed phenotype [4,7,24]. Therefore, the observed variation in Rac activity of the MDA-MB-435 cell variants is probably due to disparities in the activity of upstream regulators of Rac.

We also show that an increase in focal adhesion number per cell area correlates directly with metastatic efficiency. The physiological significance of this finding is less clear due to discrepancies in the literature on the relative contribution of focal adhesion number to cell motility. Some studies assert that a simple redistribution or relocalization of focal adhesions is sufficient to alter motility signaling pathways to the point of invasive transformation [25,26]. Other studies maintain that increased tyrosine phosphorylation and focal adhesion expression are correlated with the progression to an invasive cell phenotype [19]. Our study shows that the increased number of focal adhesions in highly metastatic cells is likely to be located in the lamellipodia, which are also increased in the more highly metastatic variants. Thus, we conclude that an increased number of focal adhesions is correlative with increased invasion. Rac activation can lead to actin polymerization and lamellipodia formation, which in turn can lead to the creation of focal adhesions. Focal adhesion formation can then activate Rac, which creates a positive feedback loop that, when disregulated, can lead to increased motility and invasion [27]. This positive feedback loop is most likely what is being activated in these cell variants to produce the specific phenotype and increased focal adhesion patterns. Supporting this hypothesis is the increased Rac activity found in the more metastatic cell variants.

Surprisingly, we show in this study that Cdc42 is not activated in any of the cell variants, including the variant with the highest metastatic efficiency. This finding is unexpected because Cdc42 is essential to cellular motility via WASP (Wiskott Aldrich Syndrome Protein), Arp2/3 (actin-related protein), and subsequent actin polymerization and filopodia formation [28]. Furthermore, Cdc42 has also been implicated in both the transformation and malignant progression of cancer [6,29]. Additionally, we show that blocking all Cdc42 activity cannot prevent the most metastatic variant from haptotaxing to basal lamina. This finding suggests that Cdc42 is not vital for malignant invasion in breast cancer, perhaps because Rac and Cdc42 are redundant in many of the roles they play in cell motility. For example, both Rac and Cdc42 can activate Arp2/3 to result in actin polymerization. Whereas Cdc42 activates Arp2/3 through WASP, Rac activates Arp2/3 through WAVE (WASP family verprolin homolog) [28].

In this study, we also demonstrate the efficacy of both the Rac1 and Rac3 isoforms in the malignant progression of human breast cancer. Because Rac1 and Rac3 both have been implicated in breast cancer [5,11,30], we carried out a comparative study between the two isoforms. We found that blocking Rac activity by expressing dominant negative mutations of Rac1 or Rac3 significantly curtailed cellular processes critical for metastatic progression *in vitro*. Moreover, we found that augmenting endogenous Rac activity by expressing dominant active Rac1 or Rac3 led to a significant increase in adhesion, migration, and invasion. Taken together, these data substantiate not only a vital role for Rac1 in cell functions relevant for breast cancer metastasis, but also a vital role for Rac3. In fact, expression of a dominant active Rac3 in the MDA-MB-435Br1 low metastatic cell variant increased invasion through basal lamina 1.5 times compared to expression of dominant active Rac1. This difference suggests an enhanced ability of the cells expressing Rac3(G12V) to invade, perhaps by a more efficient degradation of the extracellular matrix compared to the cells expressing Rac1(G12V). Because we found that expression of dominant active Rac1 or Rac3 results in a similar motile phenotype, it is possible that Rac3 is more efficient at activating proteins that degrade extracellular matrix proteins, or matrix metalloproteinases, than is Rac1.

Rac1 and Rac3 differ in their carboxyl terminus region, which is essential for subcellular localization [8]. Even though protein function is likely partially redundant due to the homology of the downstream effector loops, these proteins have been found to differ in their localization within certain types of cells [10]. Differential subcellular localization can place proteins in the proximity of different signaling cascades, resulting in differential function. More experiments are needed, however, to show that Rac1 actually acts differently to Rac3 with respect to human breast cancer.

Studies have also demonstrated that, in addition to the Rac proteins, Rho proteins, especially RhoC, may contribute to breast cancer cell invasion. RhoC was demonstrated to be overexpressed in the human inflammatory breast cancer cell line SUM 149 and transient inhibition of RhoC in inflammatory breast cancer cells by treatment with farnesyl transferase inhibitors reduced invasion and motility *in vitro*, whereas RhoC overexpression in mammary epithelial cells resulted in a significant increase in cell migration [31]. Interestingly, the most metastatic MDA-MB-435 variant demonstrated the highest Rho activity (data not shown) and future studies will include an analysis of Rho isoforms of the metastatic phenotype in breast cancer.

## Conclusion

We found a direct correlation between increased metastatic potential and increased endogenous Rac activity in a panel of metastatic human breast cancer cells that vary in their ability to form secondary metastases in an *in vivo *model. By using variants of the same cell line, we have minimized genetic variation. It remains to be investigated, however, whether or not similar results will be observed in other cell lines with different genetic backgrounds.

The research presented here establishes a direct role for Rac3 in cell functions relevant for breast cancer progression. We found that Rac3 activation alone can significantly increase *in vitro *metastatic properties in human breast cancer cells. Currently, we are testing the hypothesis that Rac3 activation alone can increase breast cancer metastasis *in vivo *by using the nude mouse model of experimental metastasis.

## Abbreviations

Arp2/3 = actin related protein 2/3; BSA = bovine serum albumin; FBS = fetal bovine serum; FITC = fluorescein isothyocianate; PBS = phosphate-buffered saline; PI = propidium iodide; WASP = Wiskott Aldrich Syndrome protein.

## Competing interests

The authors declare that they have no competing interests.

## Authors' contributions

PB characterized the morphology and motile phenotypes of MDA-MB-435 variants and the dominant active (RacGV) cell lines, carried out activity assays, generated the dominant active stable cell lines, participated in the design of the study, and drafted the manuscript. LK generated the dominant negative stable cell lines and characterized their motility phenotypes. JP provided the cell variants and participated in the coordination of the study. SD conceived of the study, participated in its design, supervised experiments, and helped to draft the manuscript.
